# Metabolic response of people with type 2 diabetes to a high protein diet

**DOI:** 10.1186/1743-7075-1-6

**Published:** 2004-09-13

**Authors:** Frank Q Nuttall, Mary C Gannon

**Affiliations:** 1 Metabolic Research Laboratory, Endocrine, Metabolism & Nutrition Section, Minneapolis VA Medical Center, Minneapolis, USA; 2 Department of Medicine, University of Minnesota, USA; 3 Department of Food Science and Nutrition, University of Minnesota, USA

## Abstract

**Background:**

One of the major interests in our laboratory has been to develop a scientific framework for dietary advice for patients with diabetes. Knowledge regarding the metabolic consequences and potential effects on health of protein in people with type 2 diabetes has been a particular interest.

**Results:**

We recently have completed a study in which dietary protein was increased from 15% to 30% of total food energy. The carbohydrate content was decreased from 55% to 40%, i.e. dietary protein replaced part of the carbohydrate. This resulted in a significant decrease in total glycohemoglobin, a decrease in postprandial glucose concentrations and a modest increase in insulin concentration. Renal function was unchanged.

Currently we also are determining the metabolic response to a diet in which the carbohydrate content is further decreased to 20% of total food energy. The %tGHb decrease was even more dramatic than with the 40% carbohydrate diet.

**Conclusion:**

From these data we conclude that increasing the protein content of the diet at the expense of carbohydrate can reduce the 24-hour integrated plasma glucose concentration, at least over a 5-week period of time. The reduction was similar to that of oral agents. Renal function was not affected significantly. Thus, increasing the protein content of the diet with a corresponding decrease in the carbohydrate content potentially is a patient empowering way of reducing the hyperglycemia present with type 2 diabetes mellitus, independent of the use of pharmaceutical agents.

## Background

Our research group has been and continues to be interested in the metabolic response of people with type 2 diabetes to macronutrients in the diet in general. More recently, we have been particularly focused on the metabolic response to a high protein diet. The reason for this is three fold: First, for several years, one of our major goals has been to develop a scientific framework for dietary advice based on sound metabolic principles. Second, we have data that suggest that an increase in dietary protein may be salutary for people with diabetes. And lastly, knowledge regarding the metabolic consequences and potential effects on health of dietary protein has lagged far behind that of dietary fats and carbohydrates.

In this paper we will focus on the concept that an increase in dietary protein may be salutary for people with diabetes, and particularly for the control of blood glucose.

## Results

The concept that an increase in dietary protein may be useful in controlling the blood glucose would appear to be counterintuitive, since amino acids derived from ingested or endogenous proteins are major net gluconeogenic substrates.

**Figure 1:**
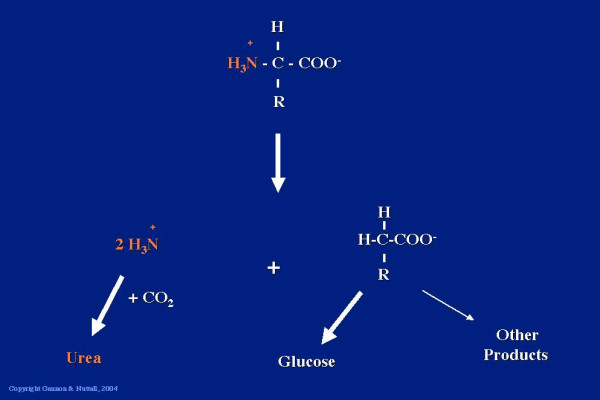
**The α amino group from an amino acid is condensed with CO_2 _to form urea. **The remaining carbon skeleton can be used to synthesize glucose.

The first step in the metabolism of amino acids is the removal of the amino group. This is condensed with CO_2 _to form urea. The remaining deaminated product is largely converted into glucose through gluconeogenesis, although a small amount is converted into other products. (Figure 1).

Indeed, in 1915, Dr. Janney [1] reported that 3.5 g glucose can be obtained from 6.25 g of ingested meat or beef protein. Thus, theoretically for every 100 g of protein ingested, 56 g of glucose can be produced. For other proteins this varies between 50 and 84 grams. Thus when developing a dietary regimen for diabetic patients, dietitians were taught to count not only carbohydrate, but also to count 56% of the protein as carbohydrate. The rationale behind this recommendation was that carbohydrates raised blood glucose, proteins are converted to glucose, therefore, dietary proteins will raise blood glucose.

However, in 1924, Dr. MacLean [2] reported that when a man with diabetes, and a fasting blood glucose of 280 mg/dl, ingested 250 g of meat, which is the equivalent of 50 grams of protein, and which should result in the production of ~25 g of glucose, there was no change in blood glucose over the 5 hours of the study. When the same subject ingested 25 g of glucose, there was a very large increase in blood glucose; indeed, it increased up to 600 mg/dl.

This lack of increase in blood glucose concentration following the ingestion of protein was confirmed by Conn and Newburgh in 1936 [3]. These investigators fed a relatively enormous amount of beef, i.e. 1.3 pounds of beef, which is the equivalent of ~136 g of protein and which should yield 68 g of glucose, to a normal subject with a fasting blood glucose of 65 mg/dl and to a subject with diabetes whose fasting blood glucose concentration was 150 mg/dl. In neither case was there an increase in blood glucose concentration over the 8 hours of this study. However, when the same subjects were given 68 g of glucose, there clearly was an increase in glucose concentration in both cases.

That ingested protein did not raise the blood glucose was largely ignored, in spite of this evidence in the scientific literature. Indeed, in his textbook in 1945 [4], Dr. Joslin, one of the most influential diabetologists at that time, was still counseling dietitians and patients to consider 56% of dietary protein as if it were carbohydrate.

### Single meal studies done in our laboratory

**Figure 2:**
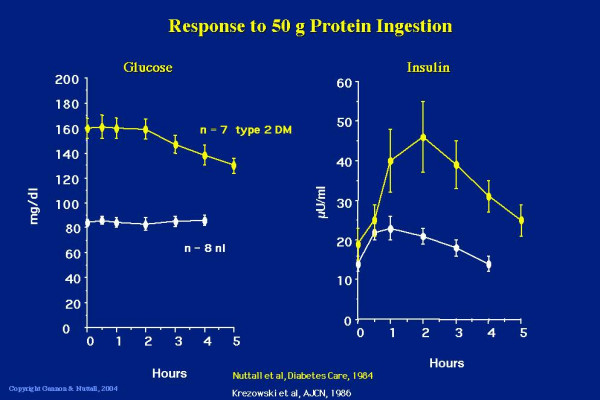
**Glucose (left panel) and insulin (right panel) response to ingestion of 50 g of protein in the form of lean beef. **Data from 8 non-diabetic subjects (white lines, bottom) and 7 subjects with type 2 diabetes (yellow lines, top). (From [5,6])

With this background information, we decided to do a study expanding on these early observations. Seven subjects with type 2 diabetes [5], and 8 subjects without diabetes [6] ingested 50 g of protein in the form of very lean beef. In the non-diabetic subjects, there was no change in blood glucose concentration over the 4 hours of the study, as had been noted previously. However, in the subjects with type 2 diabetes, the glucose concentration actually decreased over the 5 hours of that study (Figure 2).

We also determined the serum insulin response to the ingested protein and in confirmation of the studies of Berger [7], Fajans [8] and others, we observed a modest increase in the insulin concentration in the non-diabetic subjects [6]. However, there was a relatively large increase in insulin concentration in the subjects with type 2 diabetes [5]. Indeed, it was about four-fold greater than in the non-diabetic subjects (Figure 2). We also determined that the rise in insulin following the ingestion of 50 g of beef protein was just as potent in raising the insulin concentration as was the ingestion of 50 g of glucose [5]. That is, meat protein and glucose were equipotent in stimulating insulin secretion. In addition, we also demonstrated a linear dose-response relationship between the amount of beef ingested and the insulin response [5].

Since beef protein strongly stimulated insulin secretion, we next determined whether the simultaneous ingestion of protein with glucose would stimulate even more insulin and thus reduce the rise in glucose expected when glucose alone is ingested. We also were interested in determining if all common protein sources were equal in this regard. Therefore, we designed a study in which 9 – 15 males with untreated type 2 diabetes were given 50 g of glucose with or without 25 g of protein [9]. Seven protein sources were used: beef, turkey, gelatin, egg white, cottage cheese, fish and soy. The rationale behind giving 25 g of protein with 50 g of glucose, was that this ratio more closely resembles the ratio of protein to carbohydrate typically found in the diet. The plasma glucose and serum insulin concentrations were determined over a 5-hour period and the areas under the curves were calculated.

**Figure 3:**
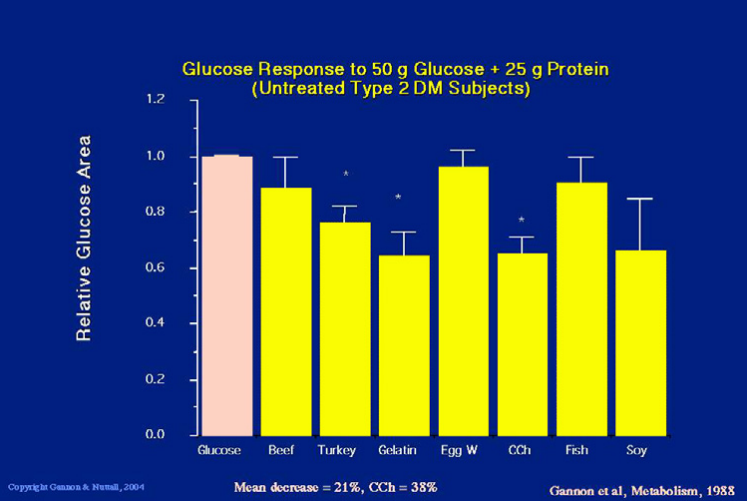
**Five hour integrated glucose area response to ingestion of 50 g glucose alone (pink bar) or 50 g glucose + 25 g protein in the form of beef, turkey, gelatin, egg white, cottage cheese, fish or soy (yellow bars, left to right)**. (From [9])

The glucose area response clearly was decreased when glucose was ingested with 25 g of protein as beef, turkey, gelatin, cottage cheese, fish and soy. Only egg white did not result in a significant decrease in glucose area response when compared to the response to ingestion of glucose alone (Figure 3).

**Figure 4:**
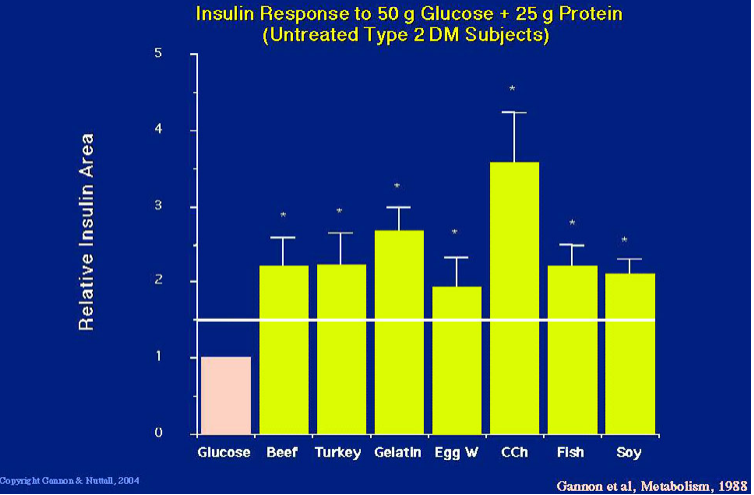
**Five hour integrated insulin area response to ingestion of 50 g glucose alone (pink bar) or 50 g glucose + 25 g protein in the form of beef, turkey, gelatin, egg white, cottage cheese, fish or soy (yellow bars, left to right). **The horizontal line indicates the expected insulin area response. (From [9])

When any of the proteins was added to the ingested glucose, the insulin area response was greatly increased (Figure 4). The smallest response was obtained with egg white, which was 190% or 1.9 fold over the response to glucose ingested alone. The greatest increase was with cottage cheese, which was 360% or 3.6 fold.

As indicated previously, beef protein, on a weight basis, was just as potent as glucose in raising the insulin concentration. Since only 25 g of beef protein was ingested in the present study, the expected response would be 150% of that observed with just glucose ingestion [5]. With beef and every other protein source studied, the insulin response was greater than the theoretical expected response (Figure 4), strongly suggesting that there is a synergistic insulin response when protein is ingested with glucose [9].

In summary, in single meal studies in people with type 2 diabetes, dietary protein strongly stimulated insulin secretion and decreased the plasma glucose response to ingested glucose.

### Insulin and Glucose Response to Mixed Meals

Based on the above observations, we decided to determine whether an increase in dietary protein in association with a decrease in carbohydrate would decrease the 24 hour integrated plasma glucose concentration, increase the 24 hour integrated insulin concentration and decrease the % total glycohemoglobin in people with type 2 diabetes ingesting mixed meals over an extended period of time.

We designed a study in which the protein content of the diet was increased from 15% of total food energy in a standard diet, to 30% protein in the experimental diet [10]. The carbohydrate content was decreased from 55% carbohydrate to 40% carbohydrate. However, it should be understood that since the additional protein can result in an increase in glucose production, the actual carbohydrate available theoretically would be about 48%, or a decrease in potential carbohydrate of only 7%. The fat content remained the same in both diets. Monounsaturated, polyunsaturated and saturated fat ratios were 10:10:10, respectively.

Twelve people with untreated type 2 diabetes were studied using a randomized, crossover design. The subjects received each diet for 5 weeks with a washout period in between. The diets were isocaloric, and all food was provided. The subjects came to the Special Diagnostic and Treatment Unit 2–3 times each week to pick up the food, to be weighed, and to provide a urine specimen for creatinine and urea nitrogen determination.

The major end-point of the study was to determine if there was a significant decrease in % total glycohemoglobin (%tGHb).

**Figure 5:**
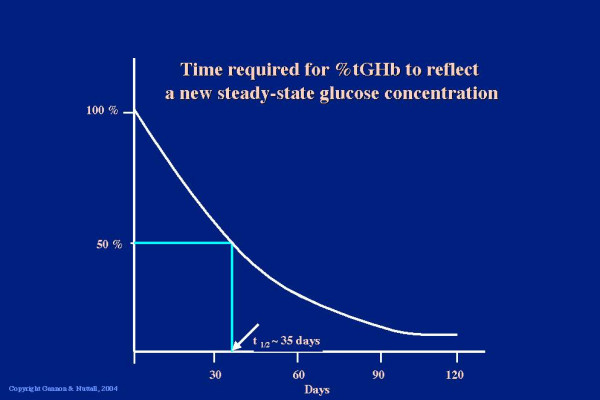
Rate of change in % tGHb

The reason that 5 weeks was chosen for the study is because this is the time required for the % total glycohemoglobin to decrease 50% of its ultimate value after a rapid stable decrease in blood glucose concentration (Figure 5), [11], i.e. the results obtained should represent 50% of the ultimate % total glycohemoglobin response.

The subjects were weight stable on both diets. We considered this to be a very important aspect of the study because we wanted to attribute any metabolic changes to the diet per se, and not to be confounded by weight loss (or gain) [10].

Urine urea, normalized to the urine creatinine, was measured as an index of compliance. Since the protein content of the diet was doubled, one would expect that the urine urea:creatinine ratio also would approximately double if the subjects were compliant. The ratio on the standard diet was ~7 and was stable throughout the 5 weeks. When the same subjects were given the 30% protein diet, the urine urea:creatinine ratio was ~13–14, i.e. a value that one would expect with good compliance with the diet.

The fasting glucose concentration did not change when the subjects received the 30% protein diet. However, the postprandial glucose concentrations were lower throughout the day [10].

**Figure 6:**
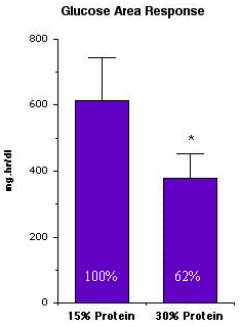
**24-hr integrated plasma glucose area response in 12 subjects with type 2 diabetes after ingesting the 15% protein or the 30% protein diet for 5 weeks. **(From [10])

Although the differences in postprandial glucose values were not very large, when integrated over the 24-hour period, there was a 38% decrease in postprandial glucose area response. If the 24-hour integrated area is considered to be 100% when the subjects ingested the 15% protein diet, when they ingested the 30% protein diet it was 62% (Figure 6).

**Figure 7:**
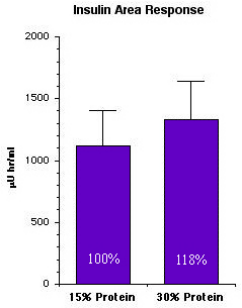
**24-hr integrated serum insulin area response in 12 subjects with type 2 diabetes after ingesting the 15% or the 30% protein diet for 5 weeks. **(From [10])

Even though the postprandial glucose concentration was decreased on the 30% protein diet, the insulin area response was modestly increased (Figure 7).

**Figure 8:**
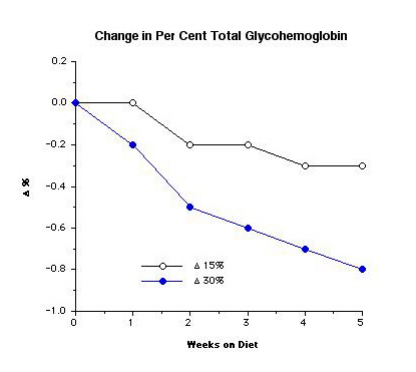
**%tGHb response in 12 subjects with type 2 diabetes at weekly intervals while ingesting a 15% or a 30% protein diet. **(From [10])

The % total glycohemoglobin decreased slightly from 8% to 7.7% during the 5 weeks of the study when the subjects were ingesting the 15% protein diet. When the subjects ingested the 30% protein diet, it decreased from 8.1 to 7.3%, i.e. the decrease was 0.8 (Figure 8).

To put this decrease in % glycohemoglobin into perspective, the Physicians Desk Reference for 2003 [12] was consulted in regard to the decrease in %HbA1c or %glycohemoglobin when subjects with type 2 diabetes were given rosiglitazone or metformin, drugs commonly used to treat people with type 2 diabetes. In subjects receiving 4 mg rosiglitazone twice a day, which is a maximal dose, the mean decrease in HbA1c was 0.7% over a 16-week period of time (Table 1). For metformin, at a maximum dose of 2500 mg daily, the decrease was 1.4% over a 29-week period.

**Table 1:** Comparison of treatment

**Agent**	**Dose**	**Duration of Treatment**	**Decrease in %tGHb or %HbA1c**
Rosiglitizone	4 mg bid	16 weeks	0.7%
Metformin	2500 mg	29 weeks	1.4%
30% Protein Diet		5 weeks	0.8% (1.6%)

PDR 2003 [12]

With the 30% protein diet, the decrease was 0.8% over the 5 weeks of our study. The ultimate decrease could be 1.6%, since at 5 weeks (35 days) the %tGHb would have decreased by only 50% of the expected final decrease (see Figure 5). Thus, the decrease would be similar to that obtained using either of the above two medications.

Since there has been concern that a high protein diet may impair renal function, the creatinine clearance was determined at the end of the period of time the subjects ingested the 15% protein diet and at the end of the period of time that the subjects ingested the 30% protein diet. There was essentially no difference. The microalbumin excretion also did not change (Table 2).

**Table 2:** Renal data

	**15% Protein Diet**	**30% Protein Diet**
Creatinine Clearance (ml/min)	122 ± 11	113 ± 27
Microalbumin (mg)	7.8 ± 1.7	7.0 ± 0.8

Also the differences in total cholesterol, HDL-cholesterol, LDL-cholesterol were not significant. The fasting triacylglycerol concentration decreased significantly when the subjects were on the 30% protein diet (Table 3).

**Table 3:** Lipid data

	**15% Protein Diet**	**30% Protein Diet**
Total Cholesterol (mg/dl)	181 ± 15	171 ± 12
HDL-Cholesterol (mg/dl)	38 ± 3	39 ± 3
LDL-Cholesterol (mg/dl)	100 ± 12	101 ± 12
Triacylglycerol (mg/dl)	199 ± 20	161 ± 21*

* P < 0.05

## Discussion

In summary, the integrated postprandial glucose area response was 38% less following ingestion of the 30% compared to the 15% protein diet. Total glycohemoglobin decreased significantly from 8.1 to 7.3% and potentially could result in a decrease to 6.5%. The integrated insulin concentration increased modestly. Renal function, LDL, HDL, and total cholesterol were unchanged. The triacylglycerol concentration decreased.

## Conclusions

From these data we conclude that increasing the protein content of the diet at the expense of carbohydrate can reduce the 24-hour integrated plasma glucose concentration, at least over a 5-week period of time. The reduction was similar to that of oral agents and renal function was not affected significantly. Thus, increasing the protein content of the diet with a corresponding decrease in the carbohydrate content potentially is a patient empowering way of reducing the hyperglycemia present in people with type 2 diabetes mellitus, independent of the use of pharmaceutical agents.

### Results of a further modification in macronutrient content

More recently we have completed study comparing an experimental diet to the standard diet, over a 5-week period of time. In the experimental diet, the protein was increased from 15% to 30% as in the above study. However, in this study the carbohydrate content was decreased from 55% to 20% of total food energy and the fat content was increased from 30% to 50%. The subjects studied were people with untreated type 2 diabetes. It was a weight maintenance diet, with a randomized crossover design. The %tGHb decrease was even more dramatic (9.8 to 7.6%) [13].

## Competing interests

None declared.

## Authors' contributions

Both authors were equally responsible for designing the experiments, evaluating the statistics, interpreting the data, writing the manuscript, and organizing the figures and tables.
